# Reducing the Soleus Stretch Reflex With Conditioning: Exploring Game- and Impedance-Based Biofeedback

**DOI:** 10.3389/fresc.2021.742030

**Published:** 2021-10-12

**Authors:** Ronald C. van 't Veld, Eline Flux, Alfred C. Schouten, Marjolein M. van der Krogt, Herman van der Kooij, Edwin H. F. van Asseldonk

**Affiliations:** ^1^Department of Biomechanical Engineering, University of Twente, Enschede, Netherlands; ^2^Department of Rehabilitation Medicine, Amsterdam Movement Sciences, Amsterdam UMC, Vrije Universiteit Amsterdam, Amsterdam, Netherlands; ^3^Department of Biomechanical Engineering, Delft University of Technology, Delft, Netherlands

**Keywords:** operant conditioning, plasticity, electromyography, gamification, system identification

## Abstract

People with spasticity, i.e., stretch hyperreflexia, have a limited functional independence and mobility. While a broad range of spasticity treatments is available, many treatments are invasive, non-specific, or temporary and might have negative side effects. Operant conditioning of the stretch reflex is a promising non-invasive paradigm with potential long-term sustained effects. Within this conditioning paradigm, seated participants have to reduce the mechanically elicited reflex response using biofeedback of reflex magnitude quantified using electromyography (EMG). Before clinical application of the conditioning paradigm, improvements are needed regarding the time-intensiveness and slow learning curve. Previous studies have shown that gamification of biofeedback can improve participant motivation and long-term engagement. Moreover, quantification of reflex magnitude for biofeedback using reflexive joint impedance may obtain similar effectiveness within fewer sessions. Nine healthy volunteers participated in the study, split in three groups. First, as a reference the “*Conventional”* group received EMG- and bar-based biofeedback similar to previous research. Second, we explored feasibility of game-based biofeedback with the “*Gaming”* group receiving EMG- and game-based biofeedback. Third, we explored feasibility of game- and impedance-based biofeedback with the “*Impedance”* group receiving impedance and game-based biofeedback. Participants completed five baseline sessions (without reflex biofeedback) and six conditioning sessions (with reflex biofeedback). Participants were instructed to reduce reflex magnitude without modulating background activity. The Conventional and Gaming groups showed feasibility of the protocol in 2 and 3 out of 3 participants, respectively. These participants achieved a significant Soleus short-latency (M1) within-session reduction in at least –15% in the 4th–6th conditioning session. None of the Impedance group participants showed any within-session decrease in Soleus reflex magnitude. The feasibility in the EMG- and game-based biofeedback calls for further research on gamification of the conditioning paradigm to obtain improved participant motivation and engagement, while achieving long-term conditioning effects. Before clinical application, the time-intensiveness and slow learning curve of the conditioning paradigm remain an open challenge.

## 1. Introduction

Spasticity is a common symptom after brain and neural injuries, like spinal cord injury, stroke, and cerebral palsy (1). Spasticity is defined as the exaggerated stretch reflex response, i.e., stretch hyperreflexia (2). Patients with spasticity are limited in functional independence and mobility and often experience substantial pain. A broad range of spasticity treatments is available, including physical therapy, oral medication, interventional procedures, and surgical treatments (3). Unfortunately, current treatments are invasive, non-specific, or temporary and might have negative side effects (3). Therefore, there is a clinical need for a non-invasive spasticity treatment with long-term sustained effect.

Operant conditioning of the reflex response is a promising, non-invasive paradigm to obtain a long-term spasticity reduction (4, 5). Within the conditioning paradigm, participants are trained to either increase (“up-condition”) or reduce (“down-condition”) the reflex response using biofeedback of reflex magnitude. Currently, paradigm feasibility has been shown for both electrical stimulation, i.e., H-reflex conditioning, mechanical stimulation, and stretch reflex conditioning, using electromyography (EMG) biofeedback of the calf muscles (6, 7). Both forms of stimulation have shown equal effectiveness during conditioning with static posture in able-bodied participants: an average –15% short-term (within-session) and –20% long-term (across-session) down-conditioning effect was obtained after 4–6 and 12–16 conditioning sessions, respectively (6, 7). From a practical, clinical perspective, the mechanical stimulation is advantageous as it yields higher participant comfort and applicability to other joints. Besides, protocols with EMG biofeedback require accurate electrode placement, checked using electrical stimulation, to ensure that conditioning effects are not due to across-session changes in electrode placement. Removing the need for accurate electrode placement checked *via* electrical stimulation would be beneficial considering home applications. Overall, before clinical application of the conditioning paradigm, improvements are needed regarding the time-intensiveness (3 session per week) and slow learning curve (at least 16 sessions).

As potential improvements for stretch reflex conditioning, we propose the use of gamification and reflexive joint impedance biofeedback. First, gamification entails the introduction of a gaming element into non-gaming situations, like rehabilitation, to make activities more pleasurable and increase long-term engagement (8, 9). Gamification can improve participant motivation in view of the possibly demotivating conditioning paradigm (10), given the long baseline measurements and slow learning curves (6, 7). Numerous studies have shown these improvements in motivation and engagement in patients with neurological conditions, such as cerebral palsy, stroke, and Parkinson's disease (11, 12). Alongside improved motivation, most game-based interventions ensure equal or even increased treatment effectiveness (10–12). However, negative effects of gamification were also reported, e.g., high levels of motivation due to gamification can distract from the primary motor learning goal and encourage undesirable compensation strategies (13). Therefore, it is important to assess whether gamification interferes with potential treatment outcomes.

Second, reflexive joint impedance biofeedback entails quantification of reflex magnitude using a mechanical-based methodology instead of the muscle-based EMG biofeedback to accelerate learning curves (14, 15). The impedance-based biofeedback disentangles the reflexive joint resistance due to the mechanical stimuli from other non-reflexive joint resistance contributions using joint torques and kinematics (16). As such, an impedance-based conditioning treatment would not require any electrodes or electrical stimulation. Previous study suggests a faster learning curve for impedance-based biofeedback, as participants were able to already modulate their reflex response after 2 sessions (15). Ludvig et al. (15) used a specific online algorithm to provide biofeedback on reflex magnitude (14). Thus, use of impedance- instead of EMG-based biofeedback can potentially improve the learning curve and practical execution.

The goal of this study is to explore the feasibility of two forms of biofeedback within the stretch reflex down-conditioning paradigm: (1) gamification of the biofeedback and (2) impedance based biofeedback. To explore feasibility, the within-session conditioning effect is investigated across six conditioning sessions. The investigation is split across three participant groups, executed in three separate phases: (1) “*Conventional”* receiving EMG- and bar-based biofeedback as in Mrachacz-Kersting et al. (7); (2) “*Gaming”* receiving EMG- and game-based biofeedback; and (3) “*Impedance”* receiving impedance- and game-based biofeedback. The use of a specific biofeedback method is considered feasible when the reference –15% within-session effect reported in previous studies can be achieved across the 4th–6th conditioning session (6, 7). Each experimental phase was only started once the previous experimental phase was evaluated as being feasible. Our study aims to open the way for stretch reflex conditioning as non-invasive spasticity treatment by introducing new biofeedback methods to make improvements regarding the time-intensiveness and slow learning curve.

## 2. Materials and Methods

### 2.1. Participants and Study Schedule

**Figure 1:**
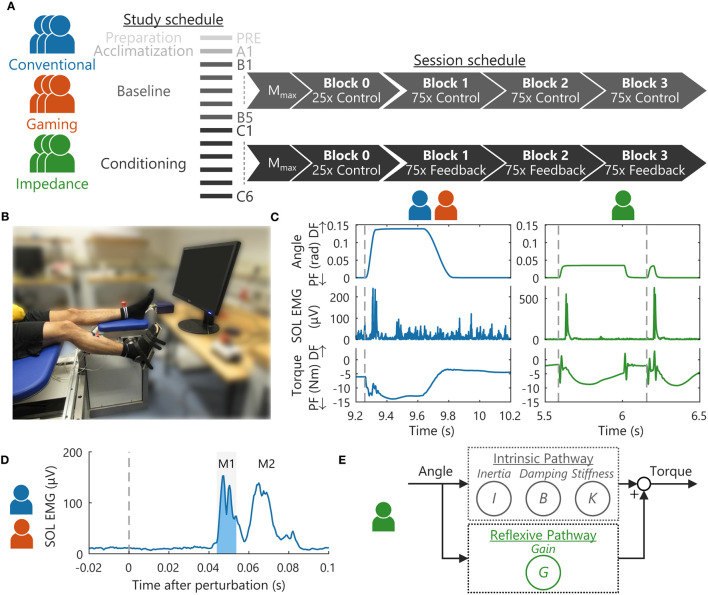
Overview experimental methodology. **(A)** Nine participants were split in three groups, all following the same 13 session study schedule (3 times per week). Per session, M_max_ was obtained using electrical stimulation, followed by 4 blocks with stretch reflexes containing either 25 or 75 feedback instances (7). **(B)** Stretch reflexes were elicited around the right ankle joint using a robotic manipulator. Participants were seated on an adjustable chair to support a static posture. **(C)** Dorsiflexion perturbations around the ankle joint elicited a stretch reflex response as visualized in the SOL muscle and torque. For the EMG-based groups, a discrete ramp-and-hold stretch profile was used (7), whereas a continuous pulse-step perturbation profile was applied for the Impedance group (14). **(D)** EMG-based groups received biofeedback on the SOL EMG, specifically background EMG activity and the short-latency (M1) reflex response *(shaded area)* (7). **(E)** The Impedance group received biofeedback on background torque and the estimated reflexive joint impedance gain (*G*). A mechanical-based methodology using recorded torques and kinematics was used to disentangle this reflexive contribution from the intrinsic contribution with parameters: inertia *I*, damping *B* and stiffness *K* (14).

Nine volunteers with no history of neuromuscular disorders participated in the study: age 26.0 ± 5.0 yr, seven women. The EEMCS/ET ethics committee of the University of Twente approved the study, and all participants provided written informed consent. The participants were split in the three biofeedback groups in order of inclusion, see Figure 1A: (1) EMG- and bar-based biofeedback (“*Conventional”*); (2) EMG- and game-based biofeedback (“*Gaming”*); and (3) impedance- and game-based biofeedback (“*Impedance”*).

All groups completed the same study schedule, designed in similar fashion to Thompson et al. (6) and Mrachacz-Kersting et al. (7), see Figure 1A. The study consisted of the following: one preparation (PRE), one acclimatization (A1), five baseline (B1-5), and six conditioning (C1-6) sessions. The preparation session was aimed at defining all personalized hardware and software settings using a protocol distinct from all other sessions. The acclimation followed the baseline session protocol and aimed to familiarize participants with this protocol (4, 6). The baseline sessions (without reflex biofeedback) and conditioning sessions (with reflex biofeedback) formed the core data collection sessions of the paradigm, see Figure 1A. Three sessions were scheduled per week (Monday, Wednesday, and Friday) with baseline and conditioning sessions typically lasting 1 h with a 1.5 h maximum. Any diurnal variation in reflexive response was minimized by scheduling all sessions at the same time of day, i.e., within the same 3 h period.

### 2.2. Experiment Setup

#### 2.2.1. Ankle Manipulator and Stretch Reflex Perturbations

Stretch reflexes were elicited around the ankle joint using a one degree-of-freedom (DOF) manipulator (Moog, Nieuw-Vennep, the Netherlands) in the sagittal plane, see Figure 1B. The manipulator applied dorsiflexion, ramp-and-hold perturbations to the right foot *via* a rigid footplate interface and Velcro straps. The encoder of the actuator of the manipulator measured foot plate angular position and velocity representing ankle angle and angular velocity. A torque sensor, located between the actuator and footplate, measured the ankle torque. Angle, velocity, and torque were recorded at 2,048 Hz, all defined positive in dorsiflexion direction. To compensate for gravitational effects on the ankle and footplate, the net torque with no voluntary participant activity was measured at the start of each block and subtracted from the torque measurements. Matlab 2017b (Mathworks, Natick, MA, USA) was used for the data collection and biofeedback during the experiment.

Participants were seated on an adjustable chair to support and control the posture during all stretch reflexes, see Figure 1B. The chair supported the upper body and upper leg to control the hip and knee angles at 120° and 150°, respectively. Both knee and hip were defined at 180° for a perfectly straight posture, and angles were measured using a goniometer. All stretch perturbations started at a 90° ankle angle, defined as the angle between shank and foot. The ankle axis of rotation was visually aligned with the actuator axis, minimizing hip and knee translations due to the applied perturbations. Participants were instructed to attain background activation by pressing into the position-controlled footplate as if rotating the ankle without use of the upper leg. Session-to-session variability of the seated posture was minimized by reusing the same personalized chair settings for each participant.

For the EMG-based groups, discrete dorsiflexion perturbations were used to elicit a stretch reflex (7). These ramp-and-hold perturbations had an 8° amplitude, 190 °/s max., velocity, 8,000 °/s^2^ max., and acceleration and 66 ms duration, see Figure 1C. Max. amplitude was held for 300 ms before the manipulator slowly returned to the 90° starting angle.

For the Impedance group, continuous dorsiflexion perturbations were used to elicit a stretch reflex (17). These ramp-and-hold perturbations had an 2° amplitude, 125 °/s max., velocity, 15,800 °/s^2^ max., and acceleration and 40 ms duration, see Figure 1C. The perturbations randomly switched between “pulses,” i.e., no hold period at max. amplitude, and “steps,” i.e., a 380 ms hold period at max. amplitude. Return toward the starting angle was with an equal and opposite profile to the dorsiflexion perturbation. The perturbation profile changes compared with the EMG-based groups were made to comply with impedance estimation procedure requirements (14).

Reflexive joint impedance was estimated using a parallel-cascade identification algorithm outlined in van 't Veld et al. (17), see Figure 1E. In short, using the recorded torques and kinematics the algorithm first estimates the intrinsic impedance parameters: inertia *I*, damping *B*, and stiffness *K*. These parameters capture the joint resistance in response to the mechanical perturbations from the tissue-related, non-neural origin, and tonic neural origin. The predicted intrinsic torque resulting from these parameters is subtracted from the total torque measured to estimate the reflexive torque. The gain *G* of the reflexive pathway is then estimated by relating this reflexive torque to the 40 ms-delayed, half-wave rectified velocity. The gain *G* reflects the joint resistance magnitude in response to the mechanical perturbations from a phasic neural origin. The parameters estimated within the initial 30 s of each block were discarded as the algorithm parameter estimation is unreliable within this transient period (14).

#### 2.2.2. Electromyography Measurements and Processing

Muscle activity was measured using the Porti EMG device (TMSi, Oldenzaal, the Netherlands). Bipolar electrodes (Kendall H124SG, 24 mm diameter; Covidien, Dublin, Ireland) were placed on the Soleus (SOL) and Tibialis Anterior (TA) according to the SENIAM guidelines (18). Session-to-session variability in electrode placement was minimized by marking each electrode on the skin (four dots on each side, re-marked every session). Moreover, a drawing of the electrode placement with respect to anatomical and skin landmarks (e.g., bones, moles, scars, and vessels) was used in case the electrode markings had faded (6, 7).

Electromyography was recorded at 2,048 Hz, high-pass filtered (2nd-order, 5 Hz, Butterworth), and rectified. SOL and TA background activity was defined as the smoothed (moving average, 100 ms window) rectified EMG (6, 7). During trials with continuous perturbations, background torque was used instead of SOL EMG, and this background activity was computed using low-pass filters (2nd-order, 0.1 Hz, Butterworth {TA}; critically damped {torque}) to reduce the influence of these perturbations (17).

Electromyography reflex magnitude was obtain using the SOL short-latency (M1) reflex response. To obtain M1 magnitude, background activity at perturbation onset was subtracted from the reflex response and the result was half-wave rectified. M1 magnitude was then defined as the root mean square (RMS) of the activity within a 10 ms window, see Figure 1D (7). This participant-specific window was manually set centered around the first peak response, typically 44–54 ms after perturbation onset, and after the last baseline sessions (B5).

#### 2.2.3. Electrical Stimulation of M_max_

To confirm correct placement of EMG electrode across-sessions, the direct motor response (M-wave) of the SOL muscle was elicited using a constant current electrical stimulator (DS7A; Digitimer, Hertfordshire, UK). The cathode (Disk electrode, 20 mm diameter; Technomed, Beek, the Netherlands) was placed in the popliteal fossa, whereas the anode (Square electrode, 41 mm height/width; Medimax Maxpatch, UK) was placed proximal to the patella. Participants were standing with a natural, upright posture for the M-wave measurements.

The simulator delivered a 1 ms width square stimulus pulse to the tibial nerve of the right leg. The M-wave magnitude was defined after each electrical stimulus as the peak-to-peak value of the unrectified SOL EMG within a 22 ms processing window (6, 7). This participant-specific window was manually placed during the preparation session, typically 4–26 ms after stimulation. To check electrode placement, the maximum M-wave M_max_ is of interest, as a steady M_max_ indicates correct electrode placement (6, 7). To obtain M_max_, stimulation intensity was gradually increased with 5 mA increments to find the intensity at which the M-wave magnitude plateaued. For data collection, three stimulation intensities above the plateau value were selected to obtain M_max_ and confirm that the intensities were within the range at which M-wave magnitude plateaued. These participant-specific intensities were set during the preparation session, e.g., at 20, 25, 30 mA or 60, 65, 70 mA.

#### 2.2.4. Intrinsic Motivation Inventory

To assess motivation and engagement, all participants completed the intrinsic motivation inventory (IMI) questionnaire after the last conditioning session (C6) (19). The questionnaire was used to assess the participant experience with the stretch reflex perturbations only, i.e., participants were instructed to ignore the electrical stimulation element for this questionnaire.

### 2.3. Experimental Protocol

#### 2.3.1. Preparation Session

All participants attended a preparation session to define all personalized hardware and software settings, retained through all other sessions (6, 7). A couple of trial electrical stimuli were applied to check whether participants felt comfortable with electrical stimulation. Two participants opted out of the study due to discomfort (lightheaded and nauseous) after these trial stimuli. New volunteers were included in the study to retain the total number of participants at nine.

To normalize EMG background activity, SOL maximum voluntary contraction (MVC) was determined (6, 7). Participants were seated (hip, knee, and ankle angle all 90°) on a stool with their upper leg locked beneath a rigid structure. Participants were instructed to produce maximum SOL activity by pressing against the rigid structure, while retaining their toes on the ground, to generate a plantarflexion torque. The SOL MVC was defined as the maximum value of the smoothed (moving average, 100 ms window) rectified SOL EMG. Each participant performed three MVC trials, and the participant-specific MVC value was set as the maximum MVC across all three trials.

To match the SOL and torque background activity target levels used throughout data collection, a tonic EMG-torque mapping was obtained. Participants executed a torque tracking task using the ankle manipulator by holding isometric torque for 3 s at 0–10 Nm in increments of 2 Nm. To obtain the EMG-torque mapping, mean SOL activity at each torque level was computed. The SOL background target was defined as a 5% MVC range matching the 4 Nm level of the EMG-torque mapping, and typical ranges were 2.5-7.5% MVC and 5–10% MVC (6, 7). The torque background target was defined as a 1 Nm range set at 3.5–4.5 Nm. The TA background activity target was set at resting level, i.e., 0–7.5 μV (6, 7). Participants completed several trials with the stretch reflex perturbations and electrical stimulation, while instructed to maintain background activity within the set targets. These trials were used to check whether participants could comfortably execute these task, given all personalized settings.

#### 2.3.2. Acclimatization, Baseline, and Conditioning Sessions

**Figure 2:**
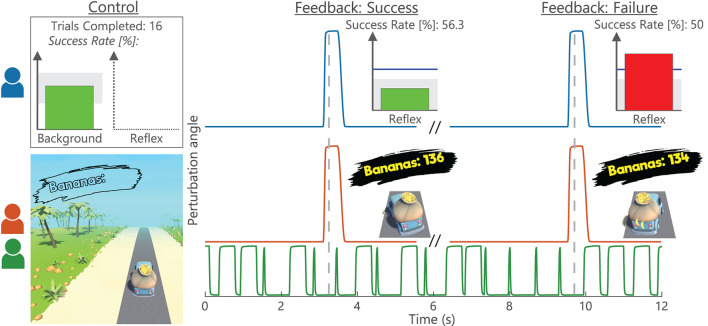
Biofeedback visualization and timing. For the (*blue*) Conventional group, a background (all trials) and a reflex (conditioning trials only) bar-graph directly represented current magnitudes. Moreover, a (*gray*) target area was displayed with the bar color visualizing whether this target was met (*green*) or not (*red*) (7). The reflex graph also showed a blue reference line based on average baseline (B1-5) reflex magnitude. The reflex biofeedback (*gray-dashed vertical)* was coupled to a stretch perturbation, displayed after a short data processing delay. Additionally, the completed number of trials and success rate were displayed. The game-based Gaming (*red*) and Impedance (*green*) groups had truck left-right position represent current background magnitude with the (*gray*) road as target area. Reflex activity controlled the number of bananas in the trunk after each feedback instance, visualized as wobble of the truck. After the wobble, all bananas were retained when the (non-visual) reflex target was met and two bananas would fall out on failure. As a result, the continuous perturbations of the Impedance group were decoupled from the feedback instances.

The acclimatization, baseline, and conditioning sessions all followed the same schedule for each participant, see Figure 1A (6, 7). For all groups, 12 electrical stimuli, i.e., four repetitions at three intensities, were applied with increasing stimulation intensity to determine M_max_. Participants were instructed to maintain steady SOL and TA background activity using bar-based biofeedback, see Figure 2 (6, 7). Stimuli were applied at 5–7 s intervals and only if participants complied with the background targets for the last 2 s.

In Block 0, the Control magnitude was measured, i.e., reflex magnitude before within-session conditioning (6, 7). Participants only received background biofeedback: SOL/TA biofeedback for EMG-based groups (6, 7) and torque/TA biofeedback for the Impedance group (15). For EMG-based groups, 25 discrete stretch perturbations were elicited at a 5–7 s interval and only if participants complied with the background activity targets for the last 2 s. For the Impedance group, these 25 discrete instances coupled to steady background activity were retained to create similar block duration across groups. Consequently, these instances were decoupled from the continuously applied pulse-step perturbation, resulting in roughly 250 stretch perturbations at a 0.5–0.7 s interval.

In Block 1–3, the Conditioned magnitude was measured, i.e., stretch reflex magnitude during within-session conditioning (6, 7). For baseline sessions, the protocol remained equal to Block 0 with only background biofeedback provided. For conditioning sessions, reflex biofeedback was added to the background biofeedback with the instruction to reduce reflex magnitude. Despite the use of continuous biofeedback by Ludvig et al. (15), the Impedance group received discrete reflex biofeedback to avoid any difficulty interpreting a biofeedback parameter with large variability (20). In each block, 75 discrete perturbations for EMG-based groups and roughly 750 continuous perturbations for the Impedance group were applied (6, 7).

### 2.4. Biofeedback

#### 2.4.1. Visualization and Timing

The Conventional group received bar-based biofeedback on background activity (all trials), and on reflex magnitude, average baseline (B1-5) reflex magnitude, number of trials completed, and success rate (conditioning trials only), see Figure 2. Biofeedback was provided *via* bar size and color, based on whether the set target was met or not. The background bar color also changed whenever TA background activity was off-target, although current TA activity was not directly visualized. Background biofeedback was continuously updated at 10 Hz, whereas the reflex biofeedback update was directly coupled to a stretch perturbation.

For game-based groups, the bar-based visualization was substituted with a third-person game about a banana delivery truck, which provided biofeedback on background activity (all trials) and reflex reduction success (conditioning trials only), see Figure 2. Reflex reduction success was represented by the number of bananas in the trunk: Starting at 150 bananas every block, two bananas would fall out after each failure to meet the reflex target at a feedback instance. An increased 30 Hz background update frequency was used for the game-based biofeedback to create a smooth gaming experience.

To obtain a pleasant gaming experience, the amount of biofeedback was reduced during gamification. As a result, participants did not receive information on the following: (1) background target success/failure; (2) quantified reflex magnitude; and (3) average baseline (B1-5) reflex magnitude, number of trials completed, and success rate. The experiment leaders could access this missing information during each block and communicate it to participants, e.g., success rates were regularly announced to the participants.

#### 2.4.2. Reward Criterion

The reflexive target range was adaptive throughout all conditioning sessions to keep the reflex reduction target equally challenging. The upper bound of the target range was set as the 66th percentile of the previous block reflex magnitude, i.e., Block 1 based on Block 0, etc. (6, 7). Participants earned a modest monetary reward if a block was completed with a success rate larger than 50%. Given the 66th percentile upper bound, a larger than 50% success rate was expected when reflex magnitude did not change between blocks (6, 7). Participants were verbally motivated to always maximize success rate, also beyond the 50% monetary threshold. Participants were not given any specific instructions or indications on reflex reduction strategies and were motivated to find their own strategy for success. Besides, participants were motivated to not purposely search for the edges of the background target ranges in order to modulate the reflex response. For additional motivation and engagement, the game-based groups also earned in-game currency per banana delivered, which could buy in-game visual upgrades for the truck and environment.

### 2.5. Data Analysis

Per session, the M-wave magnitudes were averaged across repetitions at each stimulation intensity with M_max_ defined as the maximum value across all intensities. Per stretch perturbation, background activity was computed over the 100 ms period before dorsiflexion perturbation onset for EMG-based groups (6, 7) and a shorter 40 ms period for the Impedance group to avoid movement artifacts (14). SOL and TA backgrounds were computed as mean rectified EMG and torque background as mean unfiltered torque.

The SOL M1 magnitudes, as defined in experiment setup, of both control (Block 0) and conditioned (Block 1-3) reflexes were normalized as % baseline, using baseline (B1-5) mean of the control and conditioned reflexes, respectively (6, 7). Per session, a within-session conditioning effect was defined as the mean normalized conditioned reflex minus mean normalized control reflex.

Besides, to support the use of reflexive gain *G* as biofeedback variable, the correlation between the EMG-based and impedance-based reflex magnitude was investigated. First, a set of across-block paired data points was created using the mean SOL M1 and gain *G* for each block per participant. Second, a set of within-block paired data points was created using the mean SOL M1 and gain *G* for each feedback instance per block per participant. Thus, for Block 0 (25×) and Block 1–3 (75×) all data leading up to a feedback instance were averaged for both reflexive magnitudes.

For all groups, the IMI questionnaire, taken in Session C6, consisted of four questions across four dimensions: interest-enjoyment, perceived competence, effort-importance, and tension-pressure. For each participant, all answers within a single dimension were averaged to obtain an overall score for this dimension.

### 2.6. Statistical Analysis

The feasibility of each biofeedback method was investigated by evaluating the within-session conditioning effect, with a –15% reference in Session C4-6 defined as success (6, 7). For each participant, a linear model (LM) was built using normalized SOL M1 (% baseline) as outcome measure (*N* = 2,750 for Coventional & Gaming; *N* ≈ 27,500 for Impedance). Both session (B1–C6), block (Blocks 0–3), and their interaction were used as predictor to investigate the within-session conditioning effect. Due to EMG measurement artifacts (high amplitude noise across broad frequency range), Session B1 for participant 7 and Session B5 for participant 8 were discarded. A planned contrast was used to evaluate the conditioning effect, contrasting the within-session outcome of Session C4-6 to B1-5 computed as the average of Blocks 1–3 (“Conditioned” reflex) minus Block 0 (“Control” reflex). To avoid confounding effects of the background activity, the SOL, TA, and torque background outcomes were all added to the LM as predictors to function as covariates. Per participant, the contrast was tested twice, once with and once without these covariates. Ideally, M_max_ would also be included in the LM as covariate. However, as only a single M_*max*_ outcome is available per session, adding M_max_ as covariate is impossible as this predictor would be collinear with the session predictor.

To support the need for an acclimatization session before starting the actual baseline, the SOL M1 was investigated further. An LM was built with data from Sessions A1 and B1-5 using only the mean control reflex (Block 0), using session as predictor. A planned reverse-Helmert like contrast was used to evaluate the difference in reflex magnitude between A1 vs. B1-5 and B1 vs. B2-5 for all participants combined.

The use of reflexive gain *G* as biofeedback variable was investigated using the correlation with SOL M1 magnitudes of the Impedance group (Sessions B1-C6 and Blocks 1–3). First, within-block correlation was investigated *via* a within-block Z-score standardization of all 75 data pairs for all 99 blocks (33 blocks per participants). The Z-score standardization allows to combine all data across-blocks and across-subjects before computing the correlation (17). Second, the across-block correlation was investigated by using the mean of 75 data pairs per block and using a within-subject Z-score standardization to combine data across-subjects.

## 3. Results

We explored the feasibility of three different biofeedback methods to achieve a within-session reduction of SOL M1 magnitude with a Conventional, Gaming, and Impedance group. All participants completed 12 data collection sessions: 6 acclimatization/baseline sessions (A1, B1-5) and 6 conditioning sessions (C1-6). All sessions first contained a short control block (Block 0) with 25 feedback instances followed by three blocks of 75 feedback instances without (A1–B5) or with reflex biofeedback (C1–C6). Key prerequisite on SOL M1 reduction was lack of modulation in several parameters throughout data collection to avoid confounding effects: SOL M_max_, and SOL, TA, and torque background activity.

### 3.1. Steadiness of M_max_ and Background Activity

**Figure 3:**
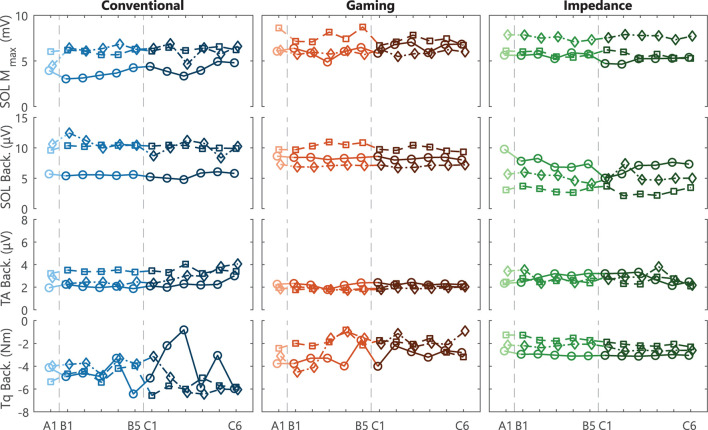
Steadiness M_max_ and background activity. Individual participant traces of SOL M_max_, and SOL, TA, and torque background activity for acclimatization (A1), baseline (B1-5), and conditioning (C1-6) sessions. All variables were required to remain steady throughout data collection. Each data point reflects the average of all blocks (Block 0–3) within a single session. Conventional and Gaming groups received biofeedback on SOL activity, whereas the Impedance group received biofeedback on torque activity. For all groups, TA activity was required to remain at a resting level (<7.5 μV). Each icon (circle, square, and diamond) per group is linked to an individual participant and consistently used across figures.

Based on session averages, all M_max_ and background activity parameters were visually considered steady throughout data collection, see Figure 3. Subsequently, steadiness of M_max_ was interpreted as consistent electrode placement throughout data collection. Similarly, steady background activity was used to avoid influences on reflex magnitude *via* voluntary increase or decrease of tonic activation. TA background also remained below resting levels indicating that co-contraction was not present. The session averages do clearly show that the EMG-based groups (Conventional and Gaming) were provided with SOL background biofeedback to keep activity steady, whereas the Impedance group used background torque biofeedback. Although no clear trends are visible, both groups show larger across-session variability for the variables on which no biofeedback was received. Thus, it was still important to evaluate the within-session effects with an LM including background variables as covariates.

### 3.2. Soleus Stretch Reflex Reduction

**Figure 4:**
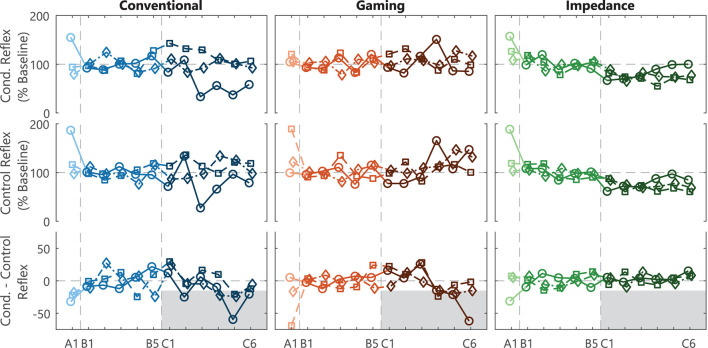
SOL M1 reflex results and within-session effect. Individual participant traces of the average conditioned reflex (mean Blocks 1-3) and control reflex (Block 0) per session for acclimatization (A1), baseline (B1-5), and conditioning (C1-6) sessions. The within-session effect is derived from the difference between the conditioned and control reflex within a session. Conventional and Gaming groups received biofeedback on SOL M1 activity, whereas the Impedance group received biofeedback on reflexive impedance gain *G*. A –15% within-session effect in session C4-6 was defined as success criteria to determine feasibility of the biofeedback method for each participant, see (*gray*) shaded target area. Each icon (circle, square, and diamond) per group is linked to an individual participant and consistently used across figures.

Both EMG-based groups (Conventional and Gaming) had several successful within-session conditioning results, reaching the reference –15% target, see bottom row Figure 4 (6, 7). Thus, within these sessions the difference between the normalized Conditioned and Control reflex measures was at least 15%, see top rows Figure 4. Contrarily, no successful within-session conditioning effect was observed for the Impedance group.

Across the full experiment, feasibility of the conditioning paradigm was confirmed in 2 (Conventional group) and 3 (Gaming group) out of 3 participants, see Table 1. In the Conventional group, the background-corrected results showed a –24% (*p* < 0.001) and –17% (*p* < 0.001) within-session effect for participants 1 and 3, whereas participant 2 showed a weaker SOL M1 reduction at –8.7% (*p* = 0.22). The Gaming group showed a –33% (*p* < 0.001), –22% (*p* < 0.001), and –16% (*p*=0.007) effect for the participants. Thus, gamification of the conditioning paradigm seemed feasible without interfering with conditioning outcomes.

**Table 1:** Contrasts between B1-5 and C4-C6 for the within-session SOL M1 effect without and with covariates.

		**LM:**~**Session** × **Block**			**LM:**~**Session** × **Block**		
					**Covariates:**~**SOL**_**back**_**+TA**_**back**_**+Torque**_**back**_		
**Group**	**Participant**	**Contrasts**	**Statistics**		**Contrasts**	**Statistics**	
Conventional	#1	–30 ± 4.3	*t*_(2, 706)_ = –6.93	*p* < 0.001	–24 ± 4.5	*t*_(2, 703)_ = –5.39	*p* < 0.001
	#2	–7.7 ± 7.0	*t*_(2, 706)_ = –1.10	*p* = 0.27	–8.7 ± 7.1	*t*_(2, 703)_ = –1.24	*p* = 0.22
	#3	–17 ± 4.2	*t*_(2, 706)_ = –4.08	*p* < 0.001	–17 ± 4.3	*t*_(2, 703)_ = –4.03	*p* < 0.001
Gaming	#4	–33 ± 7.5	*t*_(2, 706)_ = –4.36	*p* < 0.001	–33 ± 7.5	*t*_(2, 703)_ = –4.36	*p* < 0.001
	#5	–11 ± 6.5	*t*_(2, 706)_ = –1.64	*p* = 0.10	–22 ± 6.6	*t*_(2, 703)_ = –3.30	*p* < 0.001
	#6	–16 ± 6.0	*t*_(2, 706)_ = –2.72	*p* = 0.007	–16 ± 6.0	*t*_(2, 703)_ = –2.70	*p* = 0.007
Impedance	#7	4.2 ± 2.5	*t*_(24, 427)_ = 1.65	*p* = 0.10	3.4 ± 2.5	*t*_(24, 424)_ = 1.37	*p* = 0.172
	#8	5.3 ± 1.2	*t*_(25, 284)_ = 4.48	*p* < 0.001	6.3 ± 1.2	*t*_(25, 281)_ = 5.31	*p* < 0.001
	#9	2.5 ± 1.9	*t*_(27, 363)_ = 1.36	*p* = 0.17	0.29 ± 1.8	*t*_(27, 360)_ = 0.163	*p* = 0.87

*Within-session effect contrasts are expressed in % baseline, thus mean within-session effect for B1-5 equal zero within all participants. All contrasts were tested using a t-test for both the models without and with covariates*.

Feasibility was not shown for the Impedance group as all three participants showed an increase in within-session SOL M1 effect (3.4, 6.3, and 0.3%), see Table 1. Furthermore, also direct evaluation of the impedance-based reflex magnitude showed no reflex magnitude reduction (see Supplementary Figure S1). Therefore, substituting EMG- with impedance-based reflex biofeedback did not seem feasible within the conditioning paradigm.

### 3.3. Necessity Acclimatization Session

The addition of an acclimatization session before the baseline sessions was observed to potentially be beneficial for the steadiness of the reflex magnitude during baseline for all groups, see Figure 4. The results of the first depicted session (A1) could be added to the baseline session (B1-5), as the protocol executed is exactly equal. However, the reflex variables generally showed an increased control and conditioned reflexive magnitude and variability across-participants in combination with a negative within-session effect for A1 compared with B1-5. To confirm these observations, an LM of the control SOL M1 magnitude (Block 0, Session A1–B5) for all participants did indeed show a significant effect of adding the session predictor [*F*_(5, 48)_ = 5.27, p = 0.007]. A contrast further showed that the reflex magnitude for Session A1 was significantly larger than sessions B1-5 35.8 ± 7.2 % baseline [*t*_(48)_ = 4.95, *p* < 0.001]. This effect faded away when contrasting Session B1 vs. the other baseline sessions (B2-5) [*t*_(48)_ = 0.53, *p* = 0.60]. Note, no clear discrepancies between Sessions A1 and B1-5 were observed for M_max_ and all background variables, see Figure 3.

### 3.4. Correlation EMG and Impedance-Based Biofeedback

**Figure 5:**
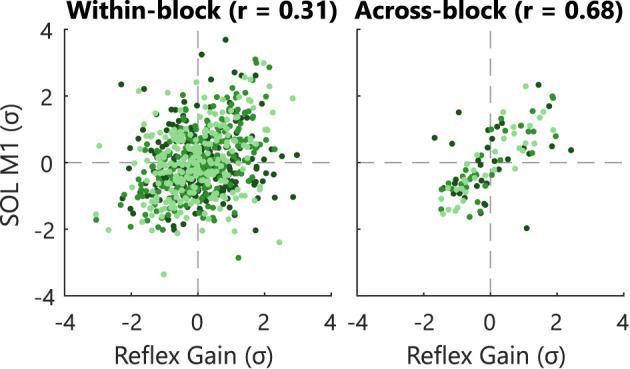
Within- and across-block correlation of reflexive biofeedback variables. Individual participants are visualized with a different color. Correlation analysis for the Impedance group for Session B1–C6 and Blocks 1–3. The within-block correlations were computed using the averaged measures per feedback instance. The across-block correlations were computed using the averaged measures per blocks. Data was Z-score standardized within-block and within-subject, respectively to allow combination of data over sessions and participants. To improve visualization only 10% of all within-block data points are shown.

The observed commonality between the EMG-based and impedance-based reflex magnitudes depended on the time frame of the evaluation, see Figure 5. A moderate correlation (*r* = 0.68) was found for the across-block correlation, whereas a weak correlation (*r* = 0.31) was found for the within-block correlation for data of all Blocks 1–3 of the Impedance groups. The moderate across-block correlation was further corroborated given the similarity between block-averaged conditioned, control, and within-session reflex outcomes, see Figure 4 and Supplementary Figure S1. Thus, the observed correlation was larger when data were averaged over a full block (ca. 750 stretches, 7.5 min) compared with averaged per feedback instance (ca. 10 stretches, 6 s).

### 3.5. Intrinsic Motivation Inventory

The IMI questionnaire showed a positive reception of the game-based conditioning paradigms, ignoring the electrical stimulation element, in terms of motivation and engagement, see Table 2. Participants in both game-based groups reported good scores for interest-enjoyment (8.5 and 8.0 out of 10) score and perceived competence (8.5 and 7.2). Note, these psychological results should be interpreted and compared with care, e.g., a large variation across the effort-importance scale was observed over the three groups, whereas no difference was expected.

**Table 2:** Intrinsic motivation inventory (IMI) scores completed after Session C6.

	**Conventional**	**Gaming**	**Impedance**
Interest-enjoyment	6.6	8.5	8.0
Competence	6.2	8.5	7.2
Effort-importance	6.8	7.0	8.6
Tension-pressure	4.5	3.3	2.1

*Scores are the across-subject averages within each group and are based on 4 questions per category. Scales used were between 1 (not at all true) and 10 (very true)*.

## 4. Discussion

The goal of this study was to explore the feasibility of two forms of biofeedback to obtain a within-session reduction of the Soleus stretch reflex with conditioning. First, we explored the feasibility of gamification and second, the feasibility of combined game- and impedance-based biofeedback. For the EMG-based groups, using either bar-based or game-based biofeedback, feasibility of the conditioning paradigm was shown in 2 and 3 out of 3 participants, respectively. Contrarily, feasibility was not shown for any participant using impedance- and game-based biofeedback. Thus, whereas the combined game- and impedance-based biofeedback was not considered feasible, the gamification of EMG-based biofeedback used to improve motivation and long-term engagement was considered feasible.

### 4.1. Feasibility Game-Based Biofeedback

Exploring the use of EMG- and game-based biofeedback within the conditioning paradigm confirmed the feasibility of the proposed biofeedback gamification. First, the switch from bar-based to game-based biofeedback did not interfere with conditioning outcomes. Our results showed feasibility of the proposed method in all participants of the Gaming group after correcting for potentially confounding background effects. Previous studies did not report on individual within-session effects and only reported a group-average –15% effect across the 16 (out of 17) successful participants, which achieved a long-term down-conditioning effect (6, 7). Comparing this result to the observed –24% Gaming group-average within-session effect should be done with caution due to the exploratory nature and small population size of our study. Moreover, the conditioned and control reflex were not interpreted separately, as previous studies showed no clear expected trends and large variability (6, 7). Second, feasibility of the gamification was also shown from a psychological perspective as the IMI scores of the Gaming group showed a positive evaluation for participant motivation and engagement. Given these results, improving motivation and long-term engagement of the conditioning paradigm to mitigate time-intensiveness and a slow learning curve is considered feasible.

Toward future use of gamification, the methodological differences between the game- (Gaming group) and bar-based (Conventional group) biofeedback were solely made to the biofeedback visualization. The main challenge toward a suitable gaming experience was the high information density of the bar-based biofeedback (6, 7). After gamification, participants most importantly did not receive information on the following: (1) background target success/failure and (2) quantified reflex magnitude. Whereas, the background biofeedback implementation has varied across previous studies on human stretch reflex reduction, all studies provided quantified reflex biofeedback (7, 21, 22). A previous study on primate stretch reflex reduction did obtain successful conditioning results without quantified reflex magnitude using food to convey success or failure (23). Our results show that such a binary (success/failure) biofeedback can also be considered feasible for human stretch reflex reduction paradigms.

### 4.2. Feasibility Combined Game- and Impedance-Based Biofeedback

Conditioning based on combined game- and impedance-based biofeedback did not yield a feasible paradigm. No participants showed a within-session reduction in reflex magnitude after impedance-based conditioning, despite positive findings in previous studies using impedance-based biofeedback outside of the conditioning paradigm (15). Any influences of potential confounders were not observed, as no trends in M_max_ or background activity were recorded and the psychometric scores for the Impedance group showed a positive evaluation. As such, accelerating the learning curve and improving practical execution of the conditioning paradigm remain an open challenge.

To find plausible explanations for the lack of within-session reflex reduction in the Impedance group, all methodological differences between Impedance and EMG-based groups were considered: (1) stretch reflex perturbations; (2) biofeedback gamification; (3) biofeedback processing; and (4) biofeedback visualization. First, compared with the EMG-based groups the stretch reflex required for the impedance-based biofeedback had a decreased amplitude, duration and velocity, whereas the acceleration and number of perturbations was increased. As expected from literature, the adapted perturbation parameters affected the reflex response as only M1 was observed, instead of both M1 and M2 (24). Yet, all previous stretch reflex studies focused on M1 conditioning (7, 21, 22), M2 does not co-condition with the M1 reflex (7), and H-reflex conditioning also just elicits a single reflexive response, most equivalent to M1 (4, 6). Therefore, the lack of M2 is not considered a plausible explanation for the lack of reflex reduction. Contrarily, the increased acceleration of the perturbation might saturate the M1 response due to the M1 acceleration dependence (24), which could plausibly explain the difficulty of reducing the reflex response. Besides, despite an increased number of perturbations, each stretch perturbation did elicit a stretch response as seen in similar impedance-based studies (14, 25). Consequently, while receiving an equal amount of feedback, participants in the Impedance group experienced an increased number of elicited reflexes, which might have influenced conditioning outcomes, although previous studies do not provide an indication whether increased perturbation occurrence would either improve or interfere with treatment outcome. Second, the gamified biofeedback visualization is not considered as likely explanation, as the exact same game was used for both Gaming and Impedance groups.

Third, an important difference between the biofeedback processing of the EMG- and impedance-based biofeedback was revealed through correlation analysis. A weak within-block correlation (*r* = 0.31) of the EMG- and impedance-based reflexive biofeedback was found based on 6 s data segments. Oppositely, for longer segments a moderate across-block correlation was found (*r* = 0.68; 7.5 min segments) and reported previously (*r* = 0.69; 60 s segments) (17). This difference between the correlation of short and long segments is likely related to the inherent 15 s risetime of the impedance estimation algorithm (14). Practically, this 15 s risetime causes a slow and delayed impedance estimation compared with the direct instance-based M1 EMG processing. Consequently, the direct coupling between a feedback instance and stretch perturbation as in the EMG-based biofeedback lacks for the impedance-based biofeedback. Fourth, the biofeedback visualization used was a mix of a continuous impedance-based (15) and discrete EMG-based paradigm (7). Ludvig et al. (15) provided continuous line-based biofeedback on magnitude, which was converted to a discrete, binary biofeedback on reflex reduction success over the last 5–7 s interval. This conversion ensured a match with the EMG-based conditioning paradigm. However, the converted impedance-based visualization did not result in a feasible paradigm, while this visualization was inspired by two previously successful studies (7, 15). This result may show the importance of quantitative or continuous impedance-based biofeedback, given the slow and delayed impedance-based biofeedback characteristics. For example, due to the variability of the reflex response, the delayed biofeedback might show reflex reduction success, while the last couple reflexes were actually too large and vice versa. Moreover, the lack of quantitative or continuous biofeedback will hide this processing effect from the participant. Overall, the lack of reflex reduction observed can potentially be explained by the delayed and decoupled biofeedback processing and its combination with the lack of a quantitative or continuous visualization.

### 4.3. Study Limitations and Future Outlook

This study can solely be interpreted as exploration of the feasibility of several biofeedback methods, given the limited number of participants. Furthermore, the protocol was limited to studying short-term (within-session) effects as long-term effects have been shown to arise after 12–16 sessions (6, 7). Within these restrictions, we recommend game-based biofeedback be implemented and tested in longer study schedules, with more participants and in a neurological population. Experimental execution should include a sufficient number of preliminary trials (at least a preparation and an acclimatization session) to ensure steadiness of baseline measurements. The goal of further exploring feasibility of the gamified conditioning paradigm is to increase participant motivation and long-term engagement during this time-intensive paradigm with a slow learning curve. Furthermore, feasibility should be explored in a neurological population before clinical implementation.

Before applying the conditioning paradigm clinically, improving the time-intensiveness and slow learning curves remains an open challenge. The implementation of impedance-based biofeedback, previously used to voluntarily modulate the reflex response, within the conditioning paradigm did not result in a feasible protocol. The impedance-based biofeedback was explored combined with the game-based biofeedback, whereas an impedance- and bar-based biofeedback group was not included. Therefore, exploring impedance- and bar-based biofeedback would be useful to provide a more direct comparison between impedance- and EMG-based biofeedback. Besides, potential improvements of the impedance-based biofeedback may lie within an improved algorithm without a 15 s risetime to avoid delayed biofeedback and directly couple the biofeedback with the current actions of the participants. Moreover, an improved impedance-based algorithm may solve the reduced correlation with EMG-based reflex magnitude for short data segments. Besides impedance-based biofeedback, other paradigm changes like conditioning during locomotion have also shown promising improvements of the slow learning curves (26).

### 4.4. Conclusions

We have shown the feasibility of EMG- and game-based biofeedback within the operant conditioning paradigm to obtain a within-session reduction in the SOL stretch reflex. Contrarily, we did not observe feasibility for the impedance- and game-based biofeedback. Stretch reflex conditioning should be applied clinically to potentially obtain a non-invasive spasticity treatment with long-term sustained effect. Before clinical application, the time-intensiveness and slow learning curve of the conditioning paradigm remain an open challenge. These results call for further research on gamification of conditioning paradigms to obtain improved participant motivation and engagement, while achieving long-term conditioning effects.

## Data Availability Statement

The original contributions presented in the study are publicly available. This data can be found here: https://doi.org/10.4121/c.5605085.

## Ethics Statement

The studies involving human participants were reviewed and approved by EEMCS/ET Ethics Committee of the University of Twente. The patients/participants provided their written informed consent to participate in this study.

## Author Contributions

RV, EF, and EA developed the experimental protocol. RV executed the experimental protocol and processed the data. RV and EF prepared the manuscript. EF, AS, HK, MK, and EA assisted with data processing and reviewed the manuscript. All authors have read and approved the final manuscript.

## Funding

This work was supported by the Netherlands Organisation for Scientific Research (NWO), domain Applied and Engineering Sciences under project number 14903 (Reflexioning project).

## Conflict of Interest

The authors declare that the research was conducted in the absence of any commercial or financial relationships that could be construed as a potential conflict of interest.

## Publisher's Note

All claims expressed in this article are solely those of the authors and do not necessarily represent those of their affiliated organizations, or those of the publisher, the editors and the reviewers. Any product that may be evaluated in this article, or claim that may be made by its manufacturer, is not guaranteed or endorsed by the publisher.
