# Supplementary material for: Reducing the Soleus Stretch Reflex With Conditioning: Exploring Game- and Impedance-Based Biofeedback
Source: Front Rehabil Sci. 2021 Oct 12;2:742030. doi: 10.3389/fresc.2021.742030 (PMC9397960; doi:10.3389/fresc.2021.742030)
Supplement: Supplementary file 1 [file Image_1.PDF]

Supplementary Material

1 SUPPLEMENTARY DATA

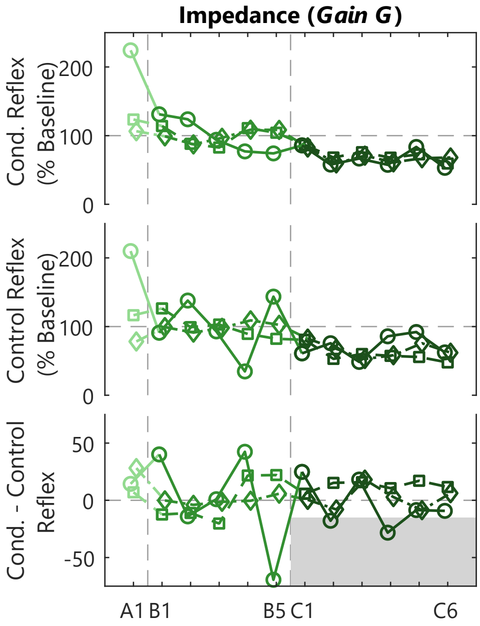

Figure S1. Reflexive impedance gain G results and within-session effect. Individual participant traces of the average conditioned reflex (mean Blocks 1-3) and control reflex (Block 0) per session for acclimatization (A1), baseline (B1-5) and conditioning (C1-6) sessions. The within-session effect is derived from the difference between the conditioned and control reflex within a session. The Impedance group received feedback on the depicted reflexive impedance gain G. A -15% within-session effect in session C4-6 was defined as success criteria to determine feasibility of the biofeedback method for each participant, see (*grey*) shaded target area. Each icon (circle, square, diamond) per group is linked to an individual participant and consistently used across figures.
